# Measuring early childhood development in multiple contexts: the internal factor structure and reliability of the early Human Capability Index in seven low and middle income countries

**DOI:** 10.1186/s12887-019-1852-5

**Published:** 2019-12-03

**Authors:** Alanna Sincovich, Tess Gregory, Cristian Zanon, Daniel D. Santos, John Lynch, Sally A. Brinkman

**Affiliations:** 1 School of Public Health, University of Adelaide, Level 9, Adelaide Health and Medical Science Building, 57 North Terrace, Adelaide, South Australia 5005 Australia; 2 Telethon Kids Institute, University of Western Australia, Level 15, 31 Flinders St, Adelaide, South Australia 5000 Australia; 3 Department of Developmental Psychology and Personality, Universidad Federal do Rio Grande do Sul, Av. Paulo Gama, 110, Bairro Farroupilha, Porto Alegre, Rio Grande do Sul 90040-060 Brazil; 4 Department of Economy, University of São Paulo, Av. Prof. Luciano Gualberto, 908, Butantã, São Paulo, 05508-010 Brazil; 5 Population Health Sciences, Bristol Medical School, University of Bristol, First Floor, 5 Tyndall Avenue, Bristol, BS8 1UD UK

**Keywords:** Child development, early Human Capability Index, Low and middle income countries, Program evaluation, Population monitoring

## Abstract

**Background:**

The fourth year of the Sustainable Development Agenda era calls for countries to continue to invest not only in interventions and policies that will promote global equity and sustainability, but also in the monitoring systems required to track progress against these targets. A more pragmatic solution to measuring children’s early development in low and middle income countries in particular, is required. This study explores the psychometric properties of the early Human Capability Index (eHCI), a population measure of holistic development for children aged 3–5 years, designed with the vision of being flexible and feasible for use in low resource and capacity settings.

**Methods:**

Utilizing data from seven low and middle income countries: Brazil (*n* = 1810), China (*n* = 11,421), Kiribati (*n* = 8339), Lao People’s Democratic Republic (*n* = 7493), Samoa (*n* = 12,191), Tonga (*n* = 6214), and Tuvalu (*n* = 549), analyses explored the internal factor structure and reliability of scores produced by the tool within each country.

**Results:**

Confirmatory factor analyses and internal consistency coefficients demonstrated that after local adaptation, translation, and different implementation methods across countries, the eHCI maintained the same factor structure of nine theoretically-based developmental domains: Physical Health, Verbal Communication, Cultural Knowledge, Social and Emotional Skills, Perseverance, Approaches to Learning, Numeracy, Reading, and Writing.

**Conclusions:**

Findings support the aims of the eHCI in being adaptable and applicable for use within a range of low and middle income countries to facilitate measurement and monitoring of children’s early development, as is required for the tracking of progress towards the Sustainable Development Agenda.

## Background

Global endorsement of the United Nations Sustainable Development Goals (SDGs), an agenda for which the healthy development of children is central [1], together with burgeoning evidence regarding the value of investing in children’s early years [2], have highlighted the need for services and supports that provide children with the opportunity not only to survive, but to developmentally thrive [3]. In turn, the creation and utilization of instruments that measure such development in children has gained momentum. The early Human Capability Index (eHCI), a population measure designed to capture the holistic development of children aged 3–5 years, represents one such effort. This paper presents preliminary evidence of the psychometric properties of scores produced by the eHCI, and highlights how the tool could make an important contribution to the task of evaluating early childhood policies and programs as well as monitoring children’s development in the early years.

### Tracking progress towards healthy child development

Ratified by United Nations member countries in 2015, the Sustainable Development Agenda specifies 17 goals and 169 targets to end poverty, mitigate inequality, and protect the planet for a better future [4]. The fourth year of the SDG era calls for countries to continue to invest not only in interventions and policies that will promote global equity and sustainability, but also in the monitoring systems required to track progress against these targets and thus identify those at risk of falling behind. Of particular relevance to early childhood development, SDG 4.2 states that by 2030, countries must ensure that all girls and boys have access to quality early childhood development, care and pre-primary education so that they are ready for primary education. To track progress against this target, countries are required to monitor (i) the percentage of children under 5 years of age who are developmentally on track in health, learning and psychosocial wellbeing, and (ii) national participation rates in early childhood education.

While many countries monitor national enrolment rates in early childhood education [5], few track the status of children’s early development. Measuring progress toward SDG 4.2 calls for population monitoring of children’s early health and development outcomes. Indeed, child development contributes to a number of SDG targets, including those related to health, gender equity, and poverty reduction, and thus global monitoring of children’s early development is key to supporting progress toward the broader Sustainable Development Agenda [6]. Faced with limitations in terms of the measurement instruments available as well as the resources and capacity required to implement such monitoring systems, tracking children’s health and development in low and middle income countries will be a challenge. In this, however, lies an important opportunity to promote and address the current obstacles associated with measuring children’s early development.

### A call for new measurement solutions

Measurement of children’s development is influenced by culture, language, and theory. What are considered important aspects of and appropriate goals for children’s development, as well as what are deemed suitable assessment techniques to capture this information, can vary considerably across cultures and contexts [7–9]. Consequently, although tools need to capture aspects of child development that are important to outcomes throughout the life course, they should also be aligned with local culture and early learning and development frameworks, so that they not only accurately reflect children’s capabilities, but also produce information relevant to local policy and practice [1, 8].

A number of measurement initiatives are currently underway to monitor children’s early development at national, regional, and global levels. Some examples include the Early Development Instrument (EDI), the Early Childhood Development Index (ECDI), the Caregiver Reported Early Development Instrument (CREDI), the International Development and Early Learning Assessment (IDELA), the East Asia-Pacific Early Chid Development Scales (EAP-ECDS), the Malawi Developmental Assessment Tool (MDAT), the Measurement of Development and Early Learning (MODEL), and the Regional Project on Child Development Indicators (PRIDI) [3]. Various characteristics of the instruments in use however, including the cost of licensing fees, the level of enumerator training required prior to administration, the time they take to administer, how they are administered, and their applicability and adaptability within different contexts, constitute considerable barriers to their utilization. This is especially the case in contexts where resources and capacity are limited. To overcome these challenges, international leaders in early childhood have called for a more pragmatic and reliable solution to measuring children’s early development in low and middle income countries in particular. It was against this background that the eHCI was developed.

### The early Human Capability Index

Designed to capture key aspects of holistic development in children aged 3–5 years, the eHCI was developed with the vision of being feasible for use in low resource and capacity settings while having the ability to capture change in children’s development over time [10]. The tool includes approximately 60 items (dependent upon country adaptation) spanning nine developmental domains (Physical Health, Verbal Communication, Cultural Knowledge, Social and Emotional Skills, Perseverance, Approaches to Learning, Numeracy, Reading, and Writing) and can be completed via adult report (e.g. by children’s caregivers, teachers, or early childhood practitioners) in less than 10 min. The eHCI requires minimal resources to be implemented; the tool is available for anyone to use free of charge, little enumerator training is required, and it can be completed quickly and easily by any adult who knows the child. Further, the tool was designed so that it can be easily adapted and utilized within diverse contexts for a range of purposes, including population monitoring, the evaluation of effects of early childhood policies and programs, as well as longitudinal studies seeking to predict children’s future capabilities.

### Development of the eHCI

The eHCI was originally developed in 2013 for the purposes of evaluating a program designed to support children and families to be better prepared for school in Tonga [11]. Consultations were undertaken to understand locally, what good child development at school entry looks like, first establishing broad areas (i.e. domains) of development and then identifying specific items within these areas. On the basis of consultations and the child development literature, a draft instrument was developed and independently reviewed by child development experts. Once translated into Tongan, stakeholders originally consulted reviewed the instrument to ensure content and face validity. Piloting was then conducted to determine respondent understanding of items, efficiency of data collection methods, if scale distributions were discriminating between children as theoretically expected, as well as any floor or ceiling effects (i.e. if items were too hard or too easy) for the targeted range of children aged 3–5 years. After revisions based on findings from the pilot as well as a final review by local stakeholders, the eHCI was implemented nationally. Exploration of the psychometric properties of scores produced by the eHCI census in Tonga demonstrated adequate discriminant validity (comparison of mean scores across children grouped by demographic characteristics met theoretical expectations, i.e. older children scored higher than younger children, girls received slightly higher scores than boys, children of more educated mothers received higher scores than those of less educated mothers) and internal scale reliability (through Rasch analysis) [10].

### Utilization of the eHCI

The eHCI has since been adapted and utilized to support a range of early childhood development projects in several low and middle income countries, predominantly across the Asia-Pacific region [12–17]. Similar to a number of tools designed to measure children’s development [18–20], development and adaptations of the eHCI in each new context were informed by a combination of both theoretical conceptualization as well as local expert consultation regarding the key aspects of children’s development that are predictive of future capabilities. Through these consultative processes, content and face validity of the instrument were established and adaptations and translations were ensured to be capturing the true intent of each item [21]. The internal factor structure of the instrument, however, is yet to be explored within multiple countries.

Evaluating the psychometric properties of scores produced by a measurement tool is fundamental for its future utilization and effectiveness [22]. An instrument measuring children’s development that lacks in reliability and validity could produce biased scores that lead to ill-informed decisions. With eHCI data now available in multiple countries, work is needed to explore the tool’s validity and reliability. An instrument with the properties of the eHCI that produces scores that are psychometrically robust and appropriate for use within diverse settings has potential for global applicability. Indeed, such a tool could better enable population monitoring of children’s development, as is required for SDG 4.2, particularly in low and middle income countries, with the ultimate goal of shaping services and policy to promote global equity of children’s health and development.

### The current study

This research is a first step in working to establish the psychometric properties of scores produced by the eHCI within different cultures and contexts. Utilizing data previously collected from seven low and middle income countries, Brazil, China, Kiribati, Lao People’s Democratic Republic (PDR), Samoa, Tonga, and Tuvalu, analyses sought to explore the internal factor structure and reliability of scores produced by the tool within each country. Findings will be used to guide recommendations regarding the reporting of eHCI results moving forward.

## Methods

### Measures

#### The early Human Capability Index

Completed by an adult who knows the child, the eHCI includes approximately 60 items (dependent upon country adaptation) measuring children’s development across nine domains: Physical Health, Verbal Communication, Cultural Knowledge, Social and Emotional Skills, Perseverance, Approaches to Learning, Numeracy, Reading, and Writing. Response options to each item are binary (“yes”/“no” or “able”/“unable”). The majority of items are positively worded so that the “yes”/“able” responses are scored as 1, and the “no”/“unable” responses are scored as 0. A small number of items (ranging from 4 in Kiribati and Lao PDR to 6 in Tonga) are negatively worded and thus are reverse scored. Individual item scores in each domain are averaged so that children receive a score for each developmental domain ranging from 0 to 1, with higher scores indicative of better development.

The eHCI underwent a local adaptation process to ensure the tool’s content and face validity in each country. Thus, although many items are similar across different adaptations of the eHCI, some items and domains differ across countries. To illustrate, the Perseverance domain is measured by the same 4 items across all adaptations of the eHCI. In contrast, the Physical Health domain varies from 2 items in Brazil, 3 items in Samoa and China, 4 items in Tonga, and 5 items in Tuvalu and Kiribati, while the Laotian version of the eHCI does not capture physical health as a result of local expert consultation. Table 1 presents the Tongan eHCI (in English) and the percentage of children for whom respondents reported yes/able for each item, while Additional file 1: Tables S1, Additional file 2: Table S2, Additional file 3: Table S3, Additional file 4: Table S4, Additional file 5: Table S5 and Additional file 6: Table S6 present the same information for remaining countries, highlighting similarities and differences between adapted versions of the instrument.

**Table 1:** Tongan eHCI items and n (%) children for whom their caregiver/teacher reported yes/able

Domain	Item	Yes/able	Missing
Physical Health	1. Is this child sickly or looked after poorly?^a^	826 (13.3)	5 (0.1)
	2. Does this child have good hygiene (i.e. always wash their hands after toileting)?	4929 (79.3)	13 (0.2)
	3. Does this child have positive habits?	4073 (65.5)	16 (0.3)
	4. Does this child know good foods from bad foods?	3710 (59.7)	25 (0.4)
Verbal Communication	1. Can this child use a group of words in talking?	5705 (91.8)	8 (0.1)
	2. Can this child converse with others?	5988 (96.3)	8 (0.1)
	3. Can this child talk about something that he/she has done?	5619 (90.4)	10 (0.2)
	4. Can this child give detail with good Tongan words?	3844 (61.9)	10 (0.2)
	5. Can this child hold an adult like conversation (for example talkative, always questioning)?	5078 (81.7)	16 (0.3)
Cultural Knowledge	1. Shows compassion, understanding and tolerance of others?	5229 (84.1)	15 (0.2)
	2. Can this child identify two culturally important foods/dishes?	5043 (81.1)	11 (0.2)
	3. Can this child identify two local plants that provide food/fruits?	4741 (76.3)	14 (0.2)
	4. Does this child show the Tongan cultural values of humility?	2892 (46.5)	21 (0.3)
	5. Does this child show the Tongan cultural values of devotion/commitment/obligation/responsibility?	2745 (44.2)	16 (0.3)
	6. Does this child show the Tongan cultural values of reciprocity in relationships?	2849 (45.8)	14 (0.2)
	7. Does this child participate in cultural routines?	4764 (76.7)	11 (0.2)
	8. Is this child able to say a short prayer?	5503 (88.5)	16 (0.3)
Social and Emotional	1. Is this child happy to share their toys and belongings?	5296 (85.2)	9 (0.1)
	2. Does this child take care of their own things?	4579 (73.7)	4 (0.1)
	3. Does this child demonstrate respect for adults?	3870 (62.3)	11 (0.2)
	4. Does this child demonstrate respect for other children?	3945 (63.5)	11 (0.2)
	5. Does this child accept responsibility for their actions?	4264 (68.6)	9 (0.1)
	6. Is this child considerate of other people’s feelings?	4274 (68.8)	6 (0.1)
	7. Does this child repeatedly do something wrong even though he/she has been told to stop?^a^	3612 (58.1)	9 (0.1)
	8. Is this child always helpful?	5445 (87.6)	6 (0.1)
	9. Is this child friendly to other children?	5644 (90.8)	9 (0.1)
	10. Does this child kick, bite or hit adults or other children?^a^	2294 (36.9)	12 (0.2)
	11. Is this child impatient?^a^	3868 (62.2)	11 (0.2)
	12. Does this child always understand the difference between acceptable and unacceptable behaviour?	4522 (72.8)	8 (0.1)
	13. Does this child follow simple directions on how to do something?	5210 (83.8)	6 (0.1)
Perseverance	1. Does this child always perform tasks independently?	4583 (73.8)	8 (0.1)
	2. Does this child always keep at a task until they are finished?	3348 (53.9)	9 (0.1)
	3. Does this child need constant reminding to finish something off?^a^	4468 (71.9)	10 (0.2)
	4. Does this child get easily distracted from a task?^a^	4664 (75.0)	14 (0.2)
Approaches to Learning	1. Does this child show more curiosity about something new in comparison to something familiar?	4976 (80.1)	10 (0.2)
	2. Does this child investigate/explore the function of a new toy/game/puzzle or object?	4900 (78.8)	9 (0.1)
	3. Is this child always wanting to learn new things?	4850 (78.0)	12 (0.2)
	4. When in an unfamiliar environment with a familiar person present, does this child feel free to explore?	3611 (58.1)	15 (0.2)
	5. Is this child diligent in their approach to a new job or task?	3840 (61.8)	19 (0.3)
Numeracy	1. Can this child recognise geometric shapes (e.g. triangle, circle, square)?	3322 (53.5)	13 (0.2)
	2. Can this child name and identify at least 3 colours?	4278 (68.8)	8 (0.1)
	3. Can this child sort and classify objects by common characteristics (e.g. shape, colour, size)?	3218 (51.8)	12 (0.2)
	4. Can this child name and recognise the symbol of all numbers from 1 to 10?	3020 (48.6)	15 (0.2)
	5. Can this child count to 10?	5053 (81.3)	7 (0.1)
	6. Can this child count to 20?	2073 (33.4)	9 (0.1)
	7. Can this child count to 100?	430 (6.9)	13 (0.2)
	8. Does this child know that a horse is taller than a dog?	4464 (71.8)	11 (0.2)
	9. Does this child know the order of the day (e.g. morning, then afternoon and then evening)?	2411 (38.8)	17 (0.3)
	10. Does this child understand the concepts of yesterday, today and tomorrow?	1812 (29.2)	14 (0.2)
	11. Does this child know that a vehicle weighs more than a cup?	4197 (67.5)	11 (0.2)
	12. Does this child know that the number 8 is bigger than the number 2?	2258 (36.3)	16 (0.3)
Reading	1. Does this child know the sounds of three letters of the alphabet? (phonics)	3932 (63.5)	16 (0.3)
	2. Can this child identify at least 3 letters of the alphabet?	3263 (52.5)	13 (0.2)
	3. Can this child identify at least 10 letters of the alphabet?	2232 (35.9)	14 (0.2)
	4. Can this child hold a book and turn the pages in the right way?	3811 (61.3)	10 (0.2)
	5. Can this child follow reading directions (i.e. left to right, top to bottom)?	1799 (28.9)	9 (0.1)
	6. Can this child read at least 4 popular words?	2263 (36.4)	13 (0.2)
Writing	1. Can this child draw something identifiable? (e.g. a stick person)	3531 (56.8)	9 (0.1)
	2. Can this child copy (trace) the shape of a letter (e.g. A, E, F)?	3392 (54.6)	5 (0.1)
	3. Can this child write their own name?	2428 (39.1)	9 (0.1)
	4. Can this child write short and simple words?	1452 (23.4)	9 (0.1)
	6. Can this child write short and simple sentences?	778 (12.5)	9 (0.1)

Note: ^a^reverse scored items

#### Demographic characteristics

In addition to measuring children’s development, the eHCI collects information about children’s demographic characteristics as well as relevant contextual information. Specifically, respondents provide information about children’s age, gender, and special needs status, and then dependent upon country adaptation, they also provide information about children’s height and weight, their mother’s highest level of education, whether the child has attended preschool, if there are reading materials (i.e. books) in the child’s home, and caregivers’ engagement in six different types of stimulating activities with their children in the home (e.g. reading a book, playing, counting etc). Variables presented in this manuscript include children’s age, gender, preschool attendance, as well as maternal education.

### Data collection procedures

Data included in this manuscript were collected from seven countries between 2013 and 17, utilizing different sampling techniques and data collection methods. Contextual information regarding data collection procedures in each country are summarized in Table 2.

**Table 2:** Data collection contexts and procedures

	Country context	Year/s	Respondent/s	Method	Data collection purpose	Sample
Brazil	Occupies a large area on the eastern coast of South America. In 2017, population was approx. 209,000 and GNI was USD8600 per capita.	2015	Preschool teachers	Pen and paper	Adaptation of the eHCI for the Brazilian context [16]	Children from one city in Southwest Brazil
China	In East Asia, the world’s most populous country. In 2017, population was approx. 1.4 billion and GNI was USD8690 per capita.	2015/16	Preschool teachers and caregivers	Pen and paper	Evaluate inequality in children’s outcomes across population groups [17]	Children from two provinces across Northern China
Kiribati	Comprised of 33 coral atolls and isles in the Central Pacific. In 2017, population was approx. 116,000 and GNI was USD3010 per capita.	2017	Preschool teachers and caregivers	Tablet	National baseline of child development to guide policy and programs [14]	Population; aimed to collect data for all children aged 3–5 years nationally
Lao PDR	A landlocked country in the Southeast of Asia. In 2017, population was approx. 6.9 million and GNI was USD2270 per capita.	2015/16	Caregivers	Tablet	Baseline data for an RCT designed to support children’s early development [12]	Children from five provinces across Northern Lao PDR
Samoa	Comprised of 2 main islands in the South Pacific. In 2017, population was approx. 196,000 and GNI was USD4090 per capita.	2016	Preschool teachers and caregivers	Tablet	National baseline of child development to guide policy and programs [13]	Population; aimed to collect data for all children aged 3–5 years nationally
Tonga	An archipelago of more than 170 South Pacific islands (36 inhabited). In 2017, population was approx. 108,000 and GNI was USD4010 per capita.	2013/14	Preschool teachers and caregivers	Pen and paper	Baseline data for an RCT designed to support children’s school readiness [10]	Population; aimed to collect data for all children aged 3–5 years nationally
Tuvalu	An island country in the South Pacific, comprising 9 small islands. In 2017, population was approx. 11,000 and GNI was USD4970 per capita.	2015	Preschool teachers and caregivers	Pen and paper	National baseline of child development to guide policy and programs [15]	Population; aimed to collect data for all children aged 3–5 years nationally

Note. *GNI* Gross National Income, *RCT* Randomized Control Trial. Population and Gross National Income figures sourced from World Bank [23]

### Participants

Characteristics of each country sample are presented in Table 3. Samples ranged in size from 549 children in Tuvalu to 12,191 in Samoa, with children ranging in age from 2 to 6 years. Though the eHCI was designed to capture the development of children aged 3–5 years, the tool has also been used to collect data on children who fall slightly outside of this age range. This is a result of varied data collection purposes across countries. For instance, in Lao PDR, 2 year olds were included in data collection as this dataset serves as the baseline measure for a randomized control trial; younger children needed to be included at baseline to ensure they also fall into midline and endline data collections in years to come. Each country sample had a relatively even split of males and females; maternal education ranged from the majority of children with mothers who had never attended school (30.2%), started (27.3%), or finished primary school in Lao PDR (29.1%), to the majority of children with mothers who had completed secondary school (42.2%) or tertiary studies in Tonga (17.8%); while the percentage of children who had attended preschool ranged from 23.2% in Lao PDR to 100.0% in Brazil.

**Table 3:** Sample descriptive characteristics

	Brazil n (%)	China n (%)	Kiribati n (%)	Lao PDR n (%)	Samoa n (%)	Tonga n (%)	Tuvalu n (%)
Child gender							
Male	853 (47.1)	5587 (48.9)	4269 (51.2)	3824 (51.0)	6293 (51.6)	3247 (52.3)	272 (49.5)
Female	855 (47.2)	5338 (46.7)	3915 (46.9)	3669 (49.0)	5898 (48.4)	2967 (47.7)	277 (50.5)
Missing	102 (5.6)	496 (4.3)	155 (1.9)	0 (0.0)	0 (0.0)	0 (0.0)	0 (0.0)
Child age							
2 years	57 (3.1)	54 (0.5)	948 (11.4)	1410 (18.8)	1159 (9.5)	13 (0.2)	1 (0.2)
3 years	571 (31.5)	3396 (29.7)	2185 (26.2)	1749 (23.3)	4377 (35.9)	1609 (25.9)	163 (29.7)
4 years	760 (42.0)	3329 (29.1)	2136 (25.6)	1867 (24.9)	4616 (37.9)	2058 (33.1)	180 (32.8)
5 years	420 (23.2)	3360 (29.4)	2013 (24.1)	1599 (21.3)	2039 (16.7)	2038 (32.8)	195 (35.5)
6 years	0 (0.0)	1226 (10.7)	173 (2.1)	16 (0.2)	0 (0.0)	443 (7.1)	7 (1.3)
Missing	2 (0.1)	56 (0.5)	884 (10.6)	852 (11.4)	0 (0.0)	53 (0.9)	3 (0.5)
Child preschool attendance							
Yes	1810 (100.0)	9159 (80.2)	7665 (91.9)	1738 (23.2)	4657 (38.2)	2701 (43.5)	498 (90.7)
No	0 (0.0)	2176 (19.1)	674 (8.1)	5755 (76.8)	7534 (61.8)	3513 (56.5)	51 (9.3)
Missing	0 (0.0)	86 (0.8)	0 (0.0)	0 (0.0)	0 (0.0)	0 (0.0)	0 (0.0)
Maternal Education							
No school	–	307 (2.7)	–	2265 (30.2)	–	–	–
Started Primary	–	1242 (10.9)	714 (8.6)	2045 (27.3)	–	–	–
Finished Primary	–	3372 (29.5)	1438 (17.2)	2182 (29.1)	222 (1.8)	81 (1.3)	47 (8.6)
Started Secondary	–	3563 (31.2)	2319 (27.8)	–	603 (4.9)	2399 (38.6)	89 (16.2)
Finished Secondary	–	1358 (11.9)	2710 (32.5)	754 (10.1)	10,037 (82.3)	2621 (42.2)	107 (19.5)
Tertiary	–	751 (6.6)	785 (9.4)	242 (3.2)	1329 (10.9)	1107 (17.8)	117 (21.3)
Missing	–	828 (7.2)	373 (4.5)	5 (0.1)	0 (0.0)	0 (0.0)	189 (34.4)
Total n	1810	11,421	8339	7493	12,191	6214	549

Note. Maternal education data were not collected in Brazil. In Samoa, Tonga, and Tuvalu, when responding to the maternal education item, respondents could select only one response option pertaining to primary school and so the proportions represented against the ‘finished primary school’ category for these countries may include a combination of children for whom their mother either started or finished primary school

### Statistical analysis

First, confirmatory factor analyses (CFAs) were conducted separately for each country to determine the fit of eHCI data to the theoretical structure of the instrument (i.e. nine developmental domains, or eight domains in the case of Lao PDR). Next, CFAs were conducted separately for children aged 3, 4, and 5 years old in each country (as this was the age range consistent across all countries) to explore any variation in fit based on children’s age. Children with missing age data were excluded from this analysis (Brazil *n* = 2, China *n* = 56, Kiribati *n* = 884, Lao PDR *n* = 852, Tonga *n* = 53, Tuvalu *n* = 3). Additional CFAs were conducted for the Lao PDR sample stratified by maternal education, to explore if fit of data to the theoretical structure of the eHCI varied by respondent’s level of education. Specifically, the sample was split into two groups: low maternal education (i.e. no school, started primary, finished primary) and high maternal education (i.e. finished secondary, tertiary) and CFAs were conducted separately for each group. Children with missing maternal education data were excluded from this analysis (*n* = 5). This analysis was conducted for the Lao PDR sample only, as it was the sole sample for which data were available on the education level of all respondents.

Goodness-of-fit indices including χ^2^ (*p* > 0.05 indicates good fit; Brown, 2006), root mean square error of approximation (RMSEA; values ≤0.06 indicate good model fit while those between 0.06 and 0.08 indicate satisfactory fit; Hu & Bentler, [24]; Yu, [25]), the Comparative Fit Index and the Tucker-Lewis Fit Index (CFI and TLI, respectively; values ≥0.95 indicate good fit while values between 0.90 and 0.95 indicate satisfactory fit; Hu & Bentler, [24]; Yu, [25]; Schreiber, Nora, Stage, Barlow, & King, [26]), as well as standardized factor loadings (≥ 0.4 considered high and thus deemed a good indicator of the underlying construct; Costello & Osborne, [27]) were used to evaluate model fit. CFAs were conducted in Mplus [28] utilizing polychoric correlation matrices and the weighted least squares mean and variance adjusted (WLSMV) estimation method, both of which are deemed most appropriate for use with binary-type data such as that of the eHCI [29–31], as well as oblique (geomin) factor rotation which assumes correlations amongst factors [31].

The internal reliability of eHCI domains was also examined for each country, which is often conducted in conjunction with factor analysis to measure how interrelated a set of items are and thus how well they, collectively, measure the underlying construct of focus [32]. Although the majority of similar research assesses internal reliability using Cronbach’s alpha [19, 20], increasingly, ordinal reliability coefficients, specifically ordinal alpha, are deemed to be more appropriate in the case of evaluating the internal reliability of scales including items with binary response options in particular [33]. As such, ordinal alpha coefficients were calculated as well as Cronbach’s alpha (≥ 0.70 deemed acceptable for both coefficients; Bland & Altman, [34]; Gadermann et al., [33]) to allow for comparison with previous research. These analyses were conducted using the package ‘psych’ in R-Studio [35].

## Results

Model fit indices yielded from a CFA for each country are presented in Table 4. Although a statistically significant χ^2^ indicates poor model fit, this test is sensitive to sample size and thus large samples are likely to yield significant χ^2^ values [24, 36]. Considering country samples were large, we instead rely more heavily on other fit indices. While χ^2^ values were statistically significant in all countries, RMSEA values indicated good model fit (i.e. ≤ 0.06) in each country with the exception of Samoa, for which the RMSEA value (0.07) indicated satisfactory fit. CFI and TLI values were ≥ 0.90 in all countries, again indicating satisfactory fit of eHCI data to the theoretical structure of the instrument (i.e. 9 developmental domains, or 8 domains in Lao PDR). Table 4 also presents model fit indices from CFAs conducted separately for 3, 4, and 5-year-old children, demonstrating relatively inconsistent results across countries. For instance, although model fit indices indicated better fit of data to the theoretical structure of the eHCI for 5-year-old children in Brazil and Samoa, slightly better fit was observed for 3-year-olds in Tonga and Tuvalu, relative to that of older children.

**Table 4:** Model fit indices from confirmatory factor analyses in each country

		X^2^ (df)	RMSEA (90% CI)	CFI	TLI
Brazil	Full sample	4595.48 (1503)	0.034 (0.033–0.035)	0.94	0.94
	3-year-olds	3772.55 (1394)	0.055 (0.053–0.057)	0.75	0.74
	4-year-olds	4413.833 (1503)	0.050 (0.049–0.052)	0.82	0.80
	5-year-olds	2157.71 (1448)	0.034 (0.031–0.037)	0.92	0.92
China	Full sample	35,272.80 (1616)	0.043 (0.042–0.043)	0.92	0.92
	3-year-olds	10,356.63 (1616)	0.040 (0.039–0.041)	0.94	0.93
	4-year-olds	12,039.11 (1616)	0.044 (0.043–0.045)	0.87	0.86
	5-year-olds	9892.98 (1616)	0.039 (0.038–0.040)	0.84	0.83
Kiribati	Full sample	28,191.32 (1979)	0.040 (0.039–0.040)	0.93	0.93
	3-year-olds	8200.71 (1979)	0.038 (0.037–0.039)	0.92	0.92
	4-year-olds	7057.17 (1682)	0.039 (0.038–0.040)	0.91	0.91
	5-year-olds	6245.19 (1979)	0.033 (0.032–0.034)	0.91	0.91
Lao PDR	Full sample	22,116.62 (1456)	0.044 (0.043–0.044)	0.90	0.90
	3-year-olds	4744.53 (1456)	0.036 (0.035–0.037)	0.87	0.86
	4-year-olds	5528.15 (1456)	0.039 (0.038–0.040)	0.86	0.85
	5-year-olds	5379.22 (1456)	0.041 (0.040–0.042)	0.89	0.89
Samoa	Full sample	89,517.11 (1674)	0.066 (0.065–0.066)	0.94	0.93
	3-year-olds	31,146.12 (1616)	0.065 (0.064–0.065)	0.93	0.93
	4-year-olds	34,712.58 (1674)	0.065 (0.065–0.066)	0.94	0.93
	5-year-olds	14,613.05 (1674)	0.062 (0.061–0.062)	0.94	0.94
Tonga	Full sample	23,936.63 (1793)	0.045 (0.044–0.045)	0.93	0.93
	3-year-olds	6172.07 (1793)	0.039 (0.038–0.040)	0.91	0.91
	4-year-olds	7724.07 (1793)	0.040 (0.039–0.041)	0.92	0.92
	5-year-olds	7839.75 (1793)	0.041 (0.040–0.042)	0.90	0.90
Tuvalu	Full sample	4144.74 (2043)	0.043 (0.041–0.045)	0.95	0.94
	3-year-olds	2540.33 (2043)	0.039 (0.033–0.043)	0.95	0.95
	`4-year-olds	2483.74 (1854)	0.043 (0.039–0.048)	0.89	0.88
	5-year-olds	2600.80 (2043)	0.037 (0.033–0.042)	0.89	0.89

Note. *p* < .001 for all models, *df* degrees of freedom, *CI* Confidence interval. Differing df across CFAs within a country highlight instances where items were dropped from the model to enable estimation of model parameters. In Brazil, read6 and write6 were dropped from the 3yo CFA, and write1 was dropped from the 5yo CFA. In Kiribati, all phys items were dropped from the 4yo CFA. In Samoa, phys2 was dropped from the 3yo CFA. In Tuvalu, phys3, phys4, and phys5 were dropped from the 4yo CFA

Standardized factor loadings yielded from full sample CFAs are presented in Table 5 for Tonga, and Additional file 7: Tables S7, Additional file 8: Table S8, Additional file 9: Table S9, Additional file 10: Table S10, Additional file 11: Table S11 and Additional file 12: Table S12 for remaining countries (factor loadings yielded from CFAs for 3, 4, and 5-year-old children are available from authors upon request). Items had factor loadings ≥0.40 across domains and countries with few exceptions. Items that form Numeracy, Reading, and Writing domains in particular had high factor loadings (≥ 0.80 on average) consistently across countries with few exceptions, while the strength of factor loadings for remaining developmental domains tended to be more varied across countries. For example, factor loadings for items in the Verbal Communication domain in China ranged from 0.52–0.90, while those in Lao PDR ranged from 0.73–0.94. Similarly, factor loadings for items in the Cultural Knowledge domain in Lao PDR ranged from 0.58–0.83, while those in Brazil ranged from 0.76–0.99. Reverse-scored items in Physical Health, Social and Emotional Skills, and Perseverance domains had weak factor loadings in all countries but Brazil. In contrast, Brazil was the only country in which some non-reverse-scored items had weak factor loadings. Specifically, Reading item 6 for which just under 2% of children were reported to be able to read complex sentences had a factor loading of 0.36, and Writing item 1 for which all but just under 2% of children were reported to be able to scribble on paper had a factor loading of 0.21.

**Table 5:** Factor loadings from confirmatory factor analysis in Tonga

	F1	F2	F3	F4	F5	F6	F7	F8	F9
Phys 1	0.10								
Phys 2	-0.73								
Phys 3	-0.69								
Phys 4	-0.82								
Comm 1		0.81							
Comm 2		0.91							
Comm 3		0.96							
Comm 4		0.85							
Comm 5		0.65							
Cult 1			0.81						
Cult 2			0.88						
Cult 3			0.88						
Cult 4			0.88						
Cult 5			0.88						
Cult 6			0.88						
Cult 7			0.66						
Cult 8			0.73						
Soc 1				0.65					
Soc 2				0.70					
Soc 3				0.88					
Soc 4				0.88					
Soc 5				0.61					
Soc 6				-0.10					
Soc 7				-0.73					
Soc 8				0.69					
Soc 9				0.65					
Soc 10				0.05					
Soc 11				-0.14					
Soc 12				0.81					
Soc 13				0.82					
Persev 1					0.76				
Persev 2					0.77				
Persev 3					-0.11				
Persev 4					-0.46				
Appr 1						0.82			
Appr 2						0.90			
Appr 3						0.90			
Appr 4						0.68			
Appr 5						0.93			
Num 1							0.86		
Num 2							0.85		
Num 3							0.86		
Num 4							0.86		
Num 5							0.79		
Num 6							0.79		
Num 7							0.76		
Num 8							0.84		
Num 9							0.85		
Num 10							0.85		
Num 11							0.84		
Num 12							0.87		
Read 1								0.77	
Read 2								0.93	
Read 3								0.92	
Read 4								0.78	
Read 5								0.89	
Read 6								0.75	
Writ 1									0.86
Writ 2									0.94
Writ 3									0.93
Writ 4									0.96
Writ 5									0.94

Note. *Phys* Physical Health, *Comm* Verbal Communication, *Cult* Cultural Knowledge, *Soc* Social and Emotional, *Persev* Perseverance, *Appr* Approaches to Learning, *Num* Numeracy, *Read* Reading, and *Writ* Writing

Table 6 presents model fit indices for CFAs for low versus high maternal education in Lao PDR, with standardized factor loadings presented in Additional file 13: Tables S13 and Additional file 14: Table S14. RMSEA, CFI and TLI values indicated better fit of eHCI data to the theoretical structure of the instrument (i.e. 8 domains in Lao PDR) when respondents had a higher level of education. Factor loadings for reverse-scored items in Social and Emotional Skills and Perseverance domains, however, were weak across both education groups.

**Table 6:** Model fit indices from confirmatory factor analyses in Lao PDR grouped by maternal education

	X^2^ (df)	RMSEA (90% CI)	CFI	TLI
Low maternal education	19188.69 (1456)	0.043 (0.043–0.044)	0.90	0.89
High maternal education	3222.03 (1456)	0.035 (0.033–0.037)	0.94	0.93

Note. *p* < .001 for all models, *df* degrees of freedom, *CI* Confidence interval

Finally, Table 7 presents internal consistency coefficients for eHCI domains in each country, demonstrating varied results across domains. The Numeracy domain had consistently high internal reliability across countries, with ordinal α ≥ .91, which is considered high [33]. Verbal Communication (ordinal α ≥ 0.87), Cultural Knowledge (ordinal α ≥ 0.79), Social and Emotional Skills (ordinal α ≥ 0.70), Approaches to Learning (ordinal α ≥ 0.86), Reading (ordinal α ≥ 0.87), and Writing (ordinal α ≥ 0.78) domains also yielded internal consistency coefficients deemed to be acceptable across countries. In contrast, the remaining two domains, Physical Health and Perseverance, demonstrated less than satisfactory internal reliability with ordinal α < 0.70 in all countries with the exception of Tuvalu and Kiribati on the Physical Health domain (ordinal α = 0.77 and 0.76, respectively) and Tuvalu (ordinal α = 0.75) and Brazil (ordinal α = 0.75) on the Perseverance domain.

**Table 7:** Internal reliability of eHCI domains

	Brazil		China		Kiribati		Lao PDR		Samoa		Tonga		Tuvalu	
	Cα	Oα	Cα	Oα	Cα	Oα	Cα	Oα	Cα	Oα	Cα	Oα	Cα	Oα
Physical Health	.20	.52	.25	.43	.61	.76	–	–	.34	.48	.47	.56	.61	.77
Verbal Communication	.67	.93	.67	.87	.78	.91	.70	.88	.78	.90	.69	.92	.73	.93
Cultural Knowledge	.52	.89	.80	.90	.84	.93	.53	.79	.89	.95	.79	.89	.82	.92
Social and Emotional	.85	.95	.71	.84	.83	.91	.73	.84	.85	.90	.50	.70	.80	.91
Perseverance	.75	.89	.48	.62	.26	.33	.15	.17	.38	.45	.23	.31	.60	.75
Approaches to Learning	.71	.91	.70	.86	.67	.86	.74	.88	.85	.94	.80	.91	.73	.90
Numeracy	.84	.94	.87	.95	.86	.94	.80	.91	.92	.97	.89	.96	.90	.97
Reading	.61	.87	.79	.90	.79	.91	.72	.91	.87	.95	.82	.92	.82	.93
Writing	.78	.87	.59	.78	.83	.94	.65	.93	.89	.96	.83	.96	.83	.94

Note. *Cα* Cronbach’s alpha, *Oα* Ordinal alpha

## Discussion

The current study presents the psychometric properties of scores produced by the eHCI in seven low and middle income countries. Results demonstrated adequate fit of eHCI data to the theoretical structure of the instrument measuring children’s development across 9 domains (or 8 domains in the case of Lao PDR). Overall, findings lend support to the aims of the eHCI in being adaptable and applicable for use within a range of low and middle income countries to facilitate measurement of children’s early development [10].

### Psychometric findings

Samples utilized in this research differed considerably across countries in terms of children’s demographic backgrounds, data collection methods and purposes, as well as sampling techniques and sizes. Although it might be argued that such differences present a challenge to exploring and comparing the validity and reliability of the eHCI within multiple contexts, this is the pragmatic nature in which the instrument was intended to be used; for a range of purposes and across varied contexts. As Yapa and Bärnighausen [37] discuss, the resource constraints that come with research in low and middle income countries are often the driving force behind creative solutions. As such, we argue that there is strength in that the eHCI was found to demonstrate a common underlying factor structure within the varied contexts in which the instrument has been implemented.

Numeracy, Reading, and Writing domains in particular were found to be working consistently across countries. Items that form these domains had high factor loadings and these scales had high internal reliability across countries. Similar results have been reported for other measures of children’s development, for instance, factor analyses of domains that constitute the EDI, a teacher-completed checklist measuring children’s holistic development in their first year of school, have demonstrated the Language and Cognitive Development domain (which captures children’s literacy and numeracy skills) to have the best fit across multiple countries [38]. Examination of items that form Numeracy, Reading, and Writing domains in the eHCI highlight that little adaptation of these items was required across countries. For instance, the Numeracy domain covers the same concepts of shape, colour, and number recognition, counting ability, and knowledge of numerical concepts such as time and weight, across countries. This might suggest these domains to be the more universal aspects of children’s development, indeed such skills have been demonstrated to be important predictors of outcomes throughout the life course [39, 40], and thus results are consistently strong across countries. Such skills are also arguably more easily observable (i.e. it is likely that a caregiver or teacher knows if a child can read or count, as opposed to whether they know if a child is always wanting to learn new things as measured in the Approaches to Learning domain, or if a child knows good from bad foods as captured in the Physical Health domain), which may also have had an influence on results.

In contrast, results across Physical Health, Verbal Communication, Cultural Knowledge, Social and Emotional Skills, Perseverance, and Approaches to Learning domains tended to demonstrate more variation across country samples. This is unsurprising considering the nature of the skills measured by these domains, including health and hygiene practices, verbal communication abilities, knowledge of culture and culturally acceptable behaviours, social interactions and emotional regulation, as well as how tasks are approached and the ability to complete them, which are aspects of development that would be considered to be more contextually and culturally specific. To illustrate, although Social and Emotional Skills was demonstrated to be one of the distinct domains that the eHCI captures within each country, variation in the strength of factor loadings of items that form this domain between Brazil and Lao PDR might be, in part, explained by cultural differences in social interactions and the expression of emotions between the two countries. Item factor loadings were lower in Lao PDR (on average around 0.65–0.70), an individualistic culture in which emotion is perceived to be experienced internally within an individual, whereas the same item loadings were higher in Brazil (on average around 0.80–0.85), a collectivist culture whereby emotions are thought to occur between people and thus are expressed openly [41]. These results could also be attributed to variation in methodological bias across countries [42]. For instance, acquiescence, the tendency to agree with statements, has been demonstrated to be more common in collectivist cultures [43]. Variation of results across countries in this way highlights the important influence of culture on the measurement of children’s development, and the need for tools to capture not only the aspects of children’s development that are important predictors of later outcomes, but to also be aligned with local culture in order to produce information that both accurately reflects children’s abilities and is relevant to local policy and practice [1, 8].

Reverse-scored items in the Physical Health, Social and Emotional, and Perseverance domains had weak factor loadings in all countries but Brazil, indicating that, compared to other items, they are poorer measures of the underlying constructs being measured by these domains. These results reflect initial findings from analyses conducted in Tonga following the development of the tool, with Rasch analyses indicating that a number of reverse-scored items did not fit the model well relative to other items [10]. It is possible that enumerators and/or respondents had difficulty in understanding and/or responding to these negatively worded items. Previous research has shown that reverse-scored items tend to load onto a factor separate to the construct they are intended to measure, that instead reflects aspects of item method [44, 45]. This was not observed in Brazil however, and this could be due to sample differences in this context relative to other countries. Specifically, Brazil was the only sample for which only preschool teachers completed the eHCI, as opposed to a combination of children’s caregivers and teachers, or caregivers only as in other samples. It might be that children’s caregivers and teachers do not respond to the eHCI in the same way, or that a minimum level of education or literacy is required to understand and respond to items. Indeed, results demonstrated better fit of data to the theoretical structure of the eHCI in Lao PDR amongst more educated caregivers who responded to the tool, compared to those less-educated. However, weak factor loadings for reverse-scored items were maintained when analyses were run separately for caregivers who had low versus high education. Together, results raise important questions regarding respondent reliability that need to be explored by future research.

When considering children’s age in determining how the instrument is operating, results were inconsistent across countries (i.e. in some countries best fit was observed for 3-year-olds and in others for 5-year-olds), and with the exception of Brazil, model fit indices did not vary in magnitude greatly, indicating that the eHCI appears to work relatively consistently across the age range of 3–5 years. Although these results provide some insight into how effective the eHCI is in measuring development across children of different ages, analyses focused on the discriminant validity of the tool, including if items capture a continuum of development for children of different ages, are needed to further explore this question.

Finally, internal reliability results for Physical Health and Perseverance domains indicated that items that form these domains, collectively, are not a good measure of these underlying constructs. It is possible that this is a result of reverse-scored items (which could be measuring constructs separate to that intended) making up a large proportion of these domains (i.e. one in two items in the Perseverance domain are reverse-scored across countries, while for the Physical Health domain this is the case for one in two items in Brazil, one in three items in Samoa and China, one in four items in Tonga, and one in five items in Tuvalu and Kiribati). Nevertheless, local adaptation of the tool in each country deemed all items important to children’s early development in their contexts, and thus it was not the intention of the current research to exclude items on the basis of psychometric results. An example is the first item in the Physical Health domain regarding children being frequently sick. Although we would not naturally assume that this item measures a child’s skills or capabilities (and subsequently it does not work well in the model), in the contexts of countries of focus whereby illness is common, it was deemed important for the eHCI to provide information regarding children’s experience of illness as one aspect of their holistic development.

### Study limitations

When interpreting results of the study, it is important to be cognizant of three limitations. First, although the majority of countries studied utilized a census approach to data collection and thus are considered nationally representative, sampling strategies employed in Brazil, China, and Lao PDR (see Table 2) posit that results may differ if eHCI data were to be collected on nationally representative samples in these countries. Next, results indicate that reverse-scored items may not be operating as intended. Beyond analyses presented in this study, however, the information required to be able to explore this further (for example, insight into respondents’ understanding of reverse-scored items) is not currently available. Finally, it is important to reiterate that demonstrating consistent internal factor structure and reliability, as has been done in this study, is not complete evidence of a valid tool and must be considered together with results from additional psychometric analyses.

### Study implications

Relative to other measures of early childhood development currently utilized, the eHCI requires minimal resources to be implemented. Initial psychometric results suggest that this has not come at the cost of the validity and reliability of the instrument. Demonstrating a consistent internal factor structure and reliability is one important aspect of the comprehensive evaluation of an instrument’s validity and reliability. Although not within the scope of the current study, additional work is underway to explore the extent to which eHCI domains can discriminate amongst children’s abilities by a range of demographic and contextual variables, are associated with scores on other measures of child development, show reliability amongst respondents, and are able to predict children’s future outcomes. A low-burden instrument that is both easily adaptable and psychometrically robust within multiple contexts in this way has potential utility internationally. Indeed, such a tool might better enable population monitoring of children’s development, as is required for the tracking of progress towards the Sustainable Development Agenda, particularly in low and middle income countries.

In terms of the reporting of and utilization of data produced by the eHCI, results suggest that eHCI data should continue to be reported across the instrument’s 9 theoretically-based developmental domains, or 8 domains in the case of Lao PDR. Reporting of children’s development across different areas of development in this way enables the identification of areas of both strength and need, and as a result can help to shape more targeted approaches to intervention or policy development. SDG 4.2 however, calls for the monitoring of children who are “developmentally on track”, a concept that, as with children’s development more broadly, is likely to vary across contexts. As such, if the eHCI is to be recommended to track progress against SDG 4.2 in future, research needs to not only work to further validate the instrument, but also determine how “developmentally on track” might be classified utilizing eHCI data.

## Conclusion

Initial psychometric results demonstrate that scores produced by the eHCI, after processes of local adaptation, translation and implementation, maintained a similar factor structure of 9 theoretically-based developmental domains (or 8 domains in the case of Lao PDR) within a range of low and middle income countries. Future research is needed to build on these results and help to determine if the eHCI is able to fulfil its purpose of being a reliable, valid, and feasible tool which can help to facilitate the evaluation of early childhood policies and programs as well as measurement and monitoring of children’s development in the early years, particularly in low and middle income countries.

## Supplementary information

**Additional file 1: Table S1.** Brazil eHCI items and n (%) children for whom their caregiver/ teacher reported yes/able.

**Additional file 2: Table S2.** China eHCI items and n (%) children for whom their caregiver/ teacher reported yes/able.

**Additional file 3: Table S3.** Kiribati eHCI items and n (%) children for whom their caregiver/ teacher reported yes/able.

**Additional file 4: Table S4.** Lao PDR eHCI items and n (%) children for whom their caregiver/ teacher reported yes/able.

**Additional file 5: Table S5.** Samoa eHCI items and n (%) children for whom their caregiver/ teacher reported yes/able.

**Additional file 6: Table S6.** Tuvalu eHCI items and n (%) children for whom their caregiver/ teacher reported yes/able.

**Additional file 7: Table S7.** Factor loadings from confirmatory factor analysis in Brazil.

**Additional file 8: Table S8**. Factor loadings from confirmatory factor analysis in China.

**Additional file 9: Table S9.** Factor loadings from confirmatory factor analysis in Kiribati.

**Additional file 10: Table S10.** Factor loadings from confirmatory factor analysis in Lao PDR.

**Additional file 11: Table S11.** Factor loadings from confirmatory factor analysis in Samoa.

**Additional file 12: Table S12.** Factor loadings from confirmatory factor analysis in Tuvalu.

**Additional file 13: Table S13.** Factor loadings from confirmatory factor analysis in Lao PDR – low maternal education.

**Additional file 14: Table S14.** Factor loadings from confirmatory factor analysis in Lao PDR – high maternal education.

## Data Availability

The datasets used and/or analysed during the current study are available from the corresponding author (AS) on reasonable request.

## Abbreviations

CFA: Confirmatory Factor Analysis
CFI: Comparative Fit Index
CREDI: Caregiver Reported Early Development Instrument
ECDI: Early Childhood Development Instrument
EDI: Early Development Instrument
EHCI: early Human Capability Index
IDELA: International Development and Early Learning Assessment
MDAT: Malawi Developmental Assessment Tool
MODEL: Measurement of Development and Early Learning
PDR: People’s Democratic Republic
PRIDI: Regional Project on Child Development Indicators
RMSEA: Root Mean Square Error of Approximation
SDG: Sustainable Development Goal
TLI: Tucker-Lewis Fit Index
WLSMV: Weighted Least Squares Mean and Variance
