# Supplementary material for: Measuring early childhood development in multiple contexts: the internal factor structure and reliability of the early Human Capability Index in seven low and middle income countries
Source: BMC Pediatr. 2019 Dec 3;19:471. doi: 10.1186/s12887-019-1852-5 (PMC6889461; doi:10.1186/s12887-019-1852-5)
Supplement: Supplementary file 1 — Additional file 1: Table S1. Brazil eHCI items and n (%) children for whom their caregiver/ teacher reported yes/able. [file 12887_2019_1852_MOESM1_ESM.docx]

**Supplementary Table 1.** Brazil eHCI items and n (%) children for whom their caregiver/teacher reported yes/able

| Domain | Item | Yes/Able | Missing |
| --- | --- | --- | --- |
| Physical  Health | 1. Is this child often sick?* | 186 (10.3) | 13 (0.7) |
|  | 1. Does this child have basic knowledge of hygiene (e.g. comes to school clean, washes hands, has a clean backpack and toothbrush)? | 1737 (96.0) | 10 (0.6) |
| Verbal Communication | 1. Can this child communicate what he/she wants with gestures (e.g. crying or pointing)? | 1776 (98.1) | 22 (1.2) |
|  | 1. Does this child understand the language spoken? | 1797 (99.3) | 3 (0.2) |
|  | 1. Can this child use words to get what he/she wants? | 1731 (95.6) | 16 (0.3) |
|  | 1. Can this child use a word or simple phrase to tell about his/her day? | 1663 (91.9) | 17 (0.9) |
|  | 1. Can this child use multiple phrases to tell about his/her day? | 1321 (73.0) | 17 (0.9) |
|  | 1. Can this child talk and listen to another in a conversation (maintain a dialogue)? | 1537 (84.9) | 24 (1.3) |
| Cultural Knowledge | 1. Can this child identify two animals? | 1781 (98.4) | 15 (0.8) |
|  | 1. Can this child identify two important types of food? | 1660 (91.7) | 15 (0.8) |
|  | 1. Can this child identify two plants that provide food/fruit? | 1020 (56.4) | 153 (8.5) |
|  | 1. Can this child sing familiar children’s songs (e.g. Happy Birthday)? | 1733 (95.7) | 19 (1.0) |
|  | 1. Can this child sing a holiday song (e.g. Christmas, or other dates or cultural events)? | 1626 (89.8) | 25 (1.4) |
| Social and Emotional | 1. Does this child share his/her toys and belongings? | 1611 (89.0) | 20 (1.1) |
|  | 1. Does this child take care of his/her own belongings? | 1656 (91.5) | 16 (0.9) |
|  | 1. Does this child show respect for adults? | 1716 (94.8) | 20 (1.1) |
|  | 1. Does this child show respect for other children? | 1635 (90.3) | 28 (1.5) |
|  | 1. Does this child accept responsibility for his/her actions? | 1462 (80.8) | 41 (2.3) |
|  | 1. Does this child have regard for the feelings of others? | 1604 (88.6) | 46 (2.5) |
|  | 1. Is this child collaborative? | 1633 (90.2) | 23 (1.3) |
|  | 1. Is this child friendly with other children? | 1647 (91.0) | 42 (2.3) |
|  | 1. Does this child kick, bite or hit adults or other children?* | 208 (11.5) | 47 (2.6) |
|  | 1. Does this child have difficulty waiting for his/her turn?* | 554 (30.6) | 22 (1.2) |
|  | 1. Does this child understand the difference between right and wrong? | 1551 (85.7) | 53 (2.9) |
|  | 1. Can this child follow simple instructions on how to do something? | 1654 (90.9) | 31 (1.7) |
| Perseverance | 1. Does this child perform tasks autonomously? | 1485 (82.0) | 35 (1.9) |
|  | 1. Does this child stick with a task until completion? | 1475 (81.5) | 26 (1.4) |
|  | 1. Does this child need to be constantly reminded to finish something?* | 485 (26.8) | 25 (1.4) |
|  | 1. Is this child easily distracted in a task?* | 643 (35.5) | 26 (1.4) |
| Approaches to Learning | 1. Does this child show more curiosity with something new than with something familiar? | 1633 (90.2) | 18 (1.0) |
|  | 1. Does this child investigate/explore a new toy, game, puzzle or object? | 1721 (95.1) | 5 (0.3) |
|  | 1. Does this child use objects in fantasy play/in using his/her own imagination? | 1713 (94.6) | 14 (0.8) |
|  | 1. Is this child interested in sports and games? | 1740 (96.1) | 17 (0.9) |
|  | 1. Does this child feel free to explore the school environment even without the presence of the teacher? | 1507 (83.3) | 34 (1.9) |
|  | 1. Does this child show interest or curiosity when dealing with a new task or activity? | 1613 (89.1) | 23 (1.3) |
| Numeracy | 1. Does this child recognize geometric shapes (e.g. triangle, circle, square)? | 1017 (56.2) | 103 (5.7) |
|  | 1. Can this child name and identify at least 3 colors? | 1538 (85.0) | 63 (3.5) |
|  | 1. Can this child sort and classify objects with common characteristics (e.g. shape, color, size)? | 1249 (69.0) | 83 (4.6) |
|  | 1. Can this child name and recognize the symbols of all the numbers from 1 to 10? | 489 (27.0) | 121 (6.7) |
|  | 1. Can this child count to 20? | 439 (24.3) | 122 (6.7) |
|  | 1. Can this child identify differences in height/size (e.g. a horse is taller than a dog)? | 1286 (71.0) | 123 (6.8) |
|  | 1. Does this child know the sequence of events in a day (e.g. breakfast, lunch, dinner and bedtime)? | 1161 (64.1) | 128 (7.1) |
|  | 1. Does this child understand the concepts of yesterday, today and tomorrow? | 523 (28.9) | 143 (7.9) |
|  | 1. Does this child have notions of weight (e.g. an elephant weighs more than a mouse)? | 1127 (62.3) | 146 (8.1) |
|  | 1. Can this child compare amounts (e.g. the number 8 is larger than the number 2)? | 401 (22.2) | 127 (7.0) |
| Reading | 1. Can this child follow directional reading (e.g. from left to right, top to bottom)? | 664 (36.7) | 119 (6.6) |
|  | 1. Can this child identify at least 3 letters of the alphabet? | 1383 (76.4) | 60 (3.3) |
|  | 1. Can this child identify at least 10 letters of the alphabet? | 831 (45.9) | 103 (5.7) |
|  | 1. Can this child recognize/identify at least 4 simple and usual words? | 290 (16.0) | 77 (4.3) |
|  | 1. Can this child identify or read complex words? | 21 (1.2) | 64 (3.5) |
|  | 1. Can this child read simple sentences? | 10 (0.6) | 53 (2.9) |
| Writing | 1. Can this child scribble on paper using a pen / pencil / crayon? | 1787 (98.7) | 21 (1.2) |
|  | 1. Can this child draw something identifiable (e.g. a stick figure)? | 1289 (71.2) | 42 (2.3) |
|  | 1. Can this child write at least 3 letters (e.g. A, B, C)? | 1056 (58.3) | 53 (2.9) |
|  | 1. Can this child write his/her name? | 905 (50.0) | 55 (3.0) |
|  | 1. Can this child write (or copy) simple words? | 586 (32.4) | 67 (3.7) |
|  | 1. Can this child write simple sentences? | 37 (2.0) | 62 (3.4) |

*Note.* * = reverse scored items.
