# Supplementary material for: Measuring early childhood development in multiple contexts: the internal factor structure and reliability of the early Human Capability Index in seven low and middle income countries
Source: BMC Pediatr. 2019 Dec 3;19:471. doi: 10.1186/s12887-019-1852-5 (PMC6889461; doi:10.1186/s12887-019-1852-5)
Supplement: Supplementary file 2 — Additional file 2: Table S2. China eHCI items and n (%) children for whom their caregiver/ teacher reported yes/able. [file 12887_2019_1852_MOESM2_ESM.docx]

**Supplementary Table 2.** China eHCI items and n (%) children for whom their caregiver/teacher reported yes/able

| Domain | Item | Yes/Able | Missing |
| --- | --- | --- | --- |
| Physical  Health | 1. Is this child frequently sickly?* | 2343 (20.5) | 112 (1.0) |
|  | 1. Does this child have good hygiene i.e. always wash their hands after toileting? | 7291 (63.8) | 128 (1.1) |
|  | 1. Does this child have a regular diet? | 9875 (86.4) | 139 (1.2) |
| Verbal Communication | 1. Can this child communicate their needs by crying or pointing? | 11055 (96.7) | 39 (0.3) |
|  | 1. Can this child understand local language? | 10101 (88.4) | 70 (0.6) |
|  | 1. Can this child use words to get their needs met? | 9473 (82.9) | 104 (0.9) |
|  | 1. Can this child tell you about their day using a single word or simple sentence? | 8035 (70.3) | 82 (0.7) |
|  | 1. Can this child tell you about their day using multiple sentences? | 10048 (87.9) | 107 (0.9) |
|  | 1. Can this child take turns speaking in a conversation? | 6380 (55.8) | 140 (1.2) |
| Cultural Knowledge | 1. Can this child show sympathy or compassion for others? | 7324 (64.1) | 197 (1.7) |
|  | 1. Can this child tolerate mistakes of others? | 6692 (58.5) | 163 (1.4) |
|  | 1. Can this child identify two culturally important food/fruits? | 8716 (76.2) | 102 (0.9) |
|  | 1. Does this child talk politely? | 9310 (81.4) | 127 (1.1) |
|  | 1. Does this child also treat the people well if those people treated him/he well? | 8557 (74.9) | 147 (1.3) |
|  | 1. Does this child demonstrate respect for adults? | 8226 (72.0) | 163 (1.4) |
|  | 1. Is this child good to his or her parents? | 8174 (71.5) | 173 (1.5) |
| Social and Emotional | 1. Is the child happy to share their toys and belongings? | 9130 (79.9) | 105 (0.9) |
|  | 1. Does this child take care of their own things? | 9395 (82.2) | 111 (1.0) |
|  | 1. Does this child demonstrate respect for other children? | 7389 (64.6) | 154 (1.3) |
|  | 1. Does this child accept responsibility for their actions? | 6321 (55.3) | 150 (1.3) |
|  | 1. Is this child considerate of other people's feelings? | 4489 (39.3) | 151 (1.3) |
|  | 1. Is this child helpful? | 7592 (66.4) | 139 (1.2) |
|  | 1. Is this child friendly to other children? | 9533 (83.4) | 142 (1.2) |
|  | 1. Does this child kick, bite or hit adults or other children?* | 3673 (32.1) | 102 (0.9) |
|  | 1. Is this child impatient?* | 7050 (61.7) | 163 (1.4) |
|  | 1. Does this child understand the difference between right and wrong? | 5388 (47.1) | 148 (1.3) |
| Perseverance | 1. Does this child perform tasks independently? | 5893 (51.6) | 168 (1.5) |
|  | 1. Does this child always keep at a task until they are finished? | 5450 (47.7) | 155 (1.4) |
|  | 1. Does this child need constant reminding to finish something off?* | 7962 (69.7) | 157 (1.4) |
|  | 1. Does this child get easily distracted from a task?* | 7578 (66.3) | 176 (1.5) |
| Approaches to Learning | 1. Does this child show more curiosity about something new in comparison to something familiar? | 10478 (91.7) | 103 (0.9) |
|  | 1. Does this child investigate/explore the function of a new toy/game/puzzle or object? | 9746 (85.3) | 110 (1.0) |
|  | 1. Does this child always want to learn something new? | 9264 (81.0) | 147 (1.3) |
|  | 1. When in an unfamiliar environment with a familiar person present, does this child feel free to explore? | 9087 (79.5) | 157 (1.4) |
|  | 1. Is this child diligent in their approach to a new job or task? | 8842 (77.4) | 178 (1.6) |
|  | 1. Does this child will to find out answer if she/he does not understand something? | 6898 (60.3) | 224 (2.0) |
| Numeracy | 1. Can this child recognise geometric shapes (e.g. triangle, circle, square)? | 8468 (74.1) | 142 (1.2) |
|  | 1. Can this child name and identify at least 3 colours? | 9231 (80.8) | 172 (1.5) |
|  | 1. Can this child sort and classify objects by common characteristics (e.g. shape, colour, size)? | 7874 (68.9) | 170 (1.5) |
|  | 1. Can this child name and recognise the symbol of all numbers from 1 to 10? | 8281 (72.4) | 139 (1.2) |
|  | 1. Can this child count to 10 without any help? | 8783 (76.8) | 148 (1.3) |
|  | 1. Can this child count to 20 without any help? | 6428 (56.2) | 135 (1.2) |
|  | 1. Can this child count to 100 without any help? | 2678 (23.4) | 169 (1.5) |
|  | 1. Does this child know that a cow is taller than a dog? | 9665 (84.6) | 185 (1.6) |
|  | 1. Does this child know the order of the day (e.g. breakfast then lunch then dinner then sleep?) | 8325 (72.8) | 187 (1.6) |
|  | 1. Does this child understand the concepts of yesterday, today and tomorrow? | 4917 (43.0) | 185 (1.6) |
|  | 1. Does this child know that an elephant weighs more than a mouse? | 8527 (74.6) | 166 (1.5) |
|  | 1. Does the child know that the number 8 is bigger than the number 2? | 6761 (59.1) | 210 (1.8) |
| Reading | 1. Can this child recognise 10 Chinese characters? | 4675 (40.9) | 185 (1.6) |
|  | 1. Can this child recognise 20 Chinese characters? | 2708 (23.7) | 177 (1.5) |
|  | 1. Can this child recognise 100 Chinese characters? | 820 (7.2) | 190 (1.7) |
|  | 1. Can this child hold one book in right way? | 8306 (72.7) | 188 (1.6) |
|  | 1. Can this child follow reading directions? (i.e. left to right, top to bottom) | 6406 (56.0) | 174 (1.5) |
|  | 1. Can this child read a book and turn pages by himself? | 7649 (66.9) | 188 (1.6) |
|  | 1. Can this child read simple sentences? | 4065 (35.6) | 189 (1.7) |
|  | 1. Can this child read complex sentences? | 2237 (19.6) | 205 (1.8) |
| Writing | 1. Can this child scribble on a page using a pen/pencil/crayon? | 4515 (39.5) | 208 (1.8) |
|  | 1. Can this child write at least 3 characters? | 4342 (38.0) | 199 (1.7) |
|  | 1. Can this child write simple sentences? | 1677 (14.7) | 211 (1.8) |

*Note.* * = reverse scored items.
