# Supplementary material for: Measuring early childhood development in multiple contexts: the internal factor structure and reliability of the early Human Capability Index in seven low and middle income countries
Source: BMC Pediatr. 2019 Dec 3;19:471. doi: 10.1186/s12887-019-1852-5 (PMC6889461; doi:10.1186/s12887-019-1852-5)
Supplement: Supplementary file 3 — Additional file 3: Table S3. Kiribati eHCI items and n (%) children for whom their caregiver/ teacher reported yes/able. [file 12887_2019_1852_MOESM3_ESM.docx]

**Supplementary Table 3.** Kiribati eHCI items and n (%) children for whom their caregiver/teacher reported yes/able

| Domain | Item | Yes/Able | | Missing |
| --- | --- | --- | --- | --- |
| Physical  Health | 1. Does the child get sick often?* | 5883 (70.5) | | 419 (5.0) |
|  | 1. Is the child practicing cleanliness and healthy living (cleans hands after toileting)? | 6330 (75.9) | | 285 (3.4) |
|  | 1. Is the child personally practicing cleanliness and healthy living on their own? | 5252 (63.0) | | 170 (2.0) |
|  | 1. Is the child careful from being hurt (e.g. burns, drowning, falls)? | 6670 (80.0) | | 192 (2.3) |
|  | 1. Does the child know the difference between good and bad food? | 5991 (71.8) | | 449 (5.4) |
| Verbal Communication | 1. The child is able to use a sequence of words. | 5620 (67.4) | | 161 (1.9) |
|  | 1. The child is able to use a simple sentence. | 5015 (60.1) | | 167 (2.0) |
|  | 1. The child is able to wait for the other person to finish speaking in a conversation, before they speak. | 4893 (58.7) | | 128 (1.5) |
|  | 1. The child is able to explain things in Kiribati. | 5404 (64.8) | | 144 (1.7) |
|  | 1. The child is able to communicate as a mature person (talkative, enquiring). | 6345 (76.1) | | 118 (1.4) |
|  | 1. The child knows their name. | 7806 (93.6) | | 102 (1.2) |
|  | 1. The child knows the name of one of their parents/guardians. | 7708 (92.4) | | 92 (1.1) |
| Cultural Knowledge | 1. The child is able to exhibit behaviours of affection, understanding and patience to others. | 6515 (78.1) | | 101 (1.2) |
|  | 1. The child is able to identify two valuable foods in Kiribati. | 5488 (65.8) | | 121 (1.5) |
|  | 1. The child is able to identify two edible plants in Kiribati. | 5743 (68.8) | | 160 (1.9) |
|  | 1. The child is able to express Kiribati behaviours and traditions (giving respect to others, being humble). | 5106 (61.2) | | 128 (1.5) |
|  | 1. The child is able to exhibit behaviours of trustworthiness and commitment to do something. | 5313 (63.7) | | 158 (1.9) |
|  | 1. The child is able to make good friendships. | 6515 (78.1) | | 140 (1.7) |
|  | 1. The child is able to join cultural and traditional way of Kiribati life (Kiribati local dance). | 5101 (61.1) | | 162 (1.9) |
|  | 1. The child is able to say a short prayer. | 6631 (79.5) | | 145 (1.7) |
| Social and Emotional | 1. The child is willing to share his toys and belongings with others. | 6501 (77.9) | | 99 (1.2) |
|  | 1. The child is able to keep his belongings very well. | 6179 (74.1) | | 112 (1.3) |
|  | 1. The child knows how to respect older people. | 4883 (58.5) | | 153 (1.8) |
|  | 1. The child knows how to respect other children. | 5346 (64.1) | | 157 (1.9) |
|  | 1. The child accepts his/her responsibilities when he/she is being instructed to carry them out. | 6725 (80.6) | | 133 (1.6) |
|  | 1. The child welcomes the opinions of others. | 6457 (77.4) | | 164 (2.0) |
|  | 1. The child does what he/she is supposed to do, or not to do. | 5446 (65.3) | | 157 (1.9) |
|  | 1. The child is willing to help others. | 6089 (73.0) | | 153 (1.8) |
|  | 1. The child communicates easily with other children. | 6786 (81.3) | | 101 (1.2) |
|  | 1. The child frequently kicks, bites, or hits older people or children.* | 3511 (42.1) | | 141 (1.7) |
|  | 1. The child can be patient long enough before receiving his/her needs. | 5760 (69.0) | | 130 (1.6) |
|  | 1. The child always knows the difference between good and bad. | 6147 (73.7) | | 144 (1.7) |
|  | 1. The child can follow simple instructions. | 7055 (84.6) | | 143 (1.7) |
| Perseverance | 1. The child can mostly do his/her work on his own. | 5512 (66.1) | | 88 (1.1) |
|  | 1. The child always completes his/her work. | 4894 (58.7) | | 106 (1.3) |
|  | 1. The child always needs to be reminded about completing what he/she was doing.* | 6369 (76.7) | | 137 (1.6) |
|  | 1. The child gets bored quickly when he/she was doing his/her job/task.* | 5671 (68.0) | | 159 (1.9) |
| Approaches to Learning | 1. The child prefers learning new ideas to familiar concepts. | 7293 (87.4) | | 118 (1.4) |
|  | 1. The child examines how a new toy works. | 7157 (85.8) | | 124 (1.5) |
|  | 1. The child always desires learning of new concepts. | 7104 (85.2) | | 150 (1.8) |
|  | 1. When the child is placed in an unfamiliar setting with a person they know, they are delighted to learn. | 3258 (39.1) | | 139 (1.7) |
|  | 1. The child is keen to learn new activities. | 5807 (69.6) | | 154 (1.8) |
| Numeracy | 1. The child is able to see shapes such as a triangle, a circle, and a square. | 6360 (76.2) | | 127 (1.5) |
|  | 1. The child is able to name and identify 3 colours or more. | 5412 (64.9) | | 136 (1.6) |
|  | 1. The child is able to sort and classify objects (such as shapes, colours and sizes). | 4947 (59.3) | | 198 (2.4) |
|  | 1. The child is able to pronounce and recognise numbers from 1 to 10. | 4955 (59.4) | | 157 (1.9) |
|  | 1. The child is able to count up to 10. | 5851 (70.1) | | 166 (2.0) |
|  | 1. The child is able to count up to 20. | 1512 (18.1) | | 174 (2.1) |
|  | 1. The child is able to count up to 100. | 398 (4.8) | | 190 (2.3) |
|  | 1. The child is aware that a dog is taller than a rat. | 6246 (74.9) | | 178 (2.1) |
|  | 1. The child is aware of the order of time in a day (morning, then afternoon then evening). | 3007 (36.0) | | 182 (2.2) |
|  | 1. The child is aware of yesterday, today and tomorrow. | 2273 (27.2) | | 198 (2.4) |
|  | 1. The child is aware that a chair is heavier than a pencil. | 6203 (74.4) | | 174 (2.1) |
|  | 1. The child is aware that number 8 is larger than number 2. | 3502 (42.0) | | 224 (2.7) |
| Reading | 1. The child knows the pronunciation of three letters in the sequence of A E I? | 6661 (79.8) | | 169 (2.0) |
|  | 1. The child is able to identify 3 letters or more in the sequence of A E I? | 5062 (60.7) | | 163 (2.0) |
|  | 1. The child is able to identify 10 letters or more in the sequence of A E I . . . ? | 2935 (35.2) | | 331 (4.0) |
|  | 1. The child is able to properly hold the book and appropriately turn its pages in the right order? | 4201 (50.4) | | 194 (2.3) |
|  | 1. The child is able to follow the right way of reading (from left to right, from top to bottom). | 2157 (25.9) | | 198 (2.4) |
|  | 1. The child is able to read 4 or more familiar words. | 3304 (39.6) | | 206 (2.5) |
| Writing | 1. The child is able to draw a picture that could be recognised (persons image). | 5692 (68.2) | 141 (1.7) | |
|  | 1. The child is able to copy or trace the outline of a letter over an already written letter. | 5349 (64.1) | 164 (2.0) | |
|  | 1. The child is able to write 3 letters or more (A E I). | 4288 (51.4) | 165 (2.0) | |
|  | 1. The child is able to write his name. | 2706 (32.4) | 177 (2.1) | |
|  | 1. The child is able to write simple words. | 2480 (29.7) | 174 (2.1) | |

*Note.* * = reverse scored items.
