# Supplementary material for: Measuring early childhood development in multiple contexts: the internal factor structure and reliability of the early Human Capability Index in seven low and middle income countries
Source: BMC Pediatr. 2019 Dec 3;19:471. doi: 10.1186/s12887-019-1852-5 (PMC6889461; doi:10.1186/s12887-019-1852-5)
Supplement: Supplementary file 4 — Additional file 4: Table S4. Lao PDR eHCI items and n (%) children for whom their caregiver/ teacher reported yes/able. [file 12887_2019_1852_MOESM4_ESM.docx]

**Supplementary Table 4.** Lao PDR eHCI items and n (%) children for whom their caregiver/teacher reported yes/able

| Domain | Item | Yes/Able | Missing |
| --- | --- | --- | --- |
| Verbal Communication | 1. Can child tell you what he/she wants? | 7154 (95.5) | 0 (0.0) |
|  | 1. Can child speak a few simple words or sentences to explain what happened to him/her? | 6713 (89.6) | 0 (0.0) |
|  | 1. Can child speak many words or sentences to explain what happened to him/her? | 5671 (75.7) | 0 (0.0) |
|  | 1. Can child communicate well with you on any topics? | 5708 (76.2) | 0 (0.0) |
| Cultural Knowledge | 1. Can child tell a type of animal, at least two animals in the area? | 7184 (95.9) | 0 (0.0) |
|  | 1. Can child tell a food name, at least two dishes that are available in the area? | 6695 (89.4) | 0 (0.0) |
|  | 1. Can child tell a name of a plant/vegetable/fruit, at least two types in the area? | 6760 (90.2) | 0 (0.0) |
|  | 1. Can child sing? | 3976 (53.1) | 0 (0.0) |
|  | 1. Can child participate in traditional events such as giving alms and seeing monks? | 1937 (25.9) | 0 (0.0) |
| Social and Emotional | 1. Is child happy to share his/her toys with others? | 5874 (78.4) | 0 (0.0) |
|  | 1. Does child know how to take care of his/her belongings? | 4950 (66.1) | 0 (0.0) |
|  | 1. Has child shown respect to elders? | 1907 (25.5) | 0 (0.0) |
|  | 1. Does child respect other kids? | 2800 (37.4) | 0 (0.0) |
|  | 1. Is child responsible for his/her own behaviour? | 1844 (24.6) | 0 (0.0) |
|  | 1. Does child consider other people’s feelings? | 1560 (20.8) | 0 (0.0) |
|  | 1. Can child help other people? | 3104 (41.4) | 0 (0.0) |
|  | 1. Is child friendly to other kids? | 6375 (85.1) | 0 (0.0) |
|  | 1. Is child hot tempered?* | 4141 (55.3) | 0 (0.0) |
|  | 1. Can child understand the difference between right and wrong? | 2291 (30.6) | 0 (0.0) |
|  | 1. Can child respond to a simple instruction? | 6376 (85.1) | 0 (0.0) |
|  | 1. Is your child very clingy (i.e. does not want to leave their parent’s side)?* | 6502 (86.8) | 0 (0.0) |
|  | 1. Does your child understand his/her feelings and is able to describe his/her feelings, for example by saying “I’m happy…: or “ I’m sad…”? | 2935 (39.2) | 0 (0.0) |
|  | 1. Does your child stop an activity when told to do so straight away? | 6243 (83.3) | 0 (0.0) |
| Perseverance | 1. Can child do something on his/her own? | 4791 (63.9) | 0 (0.0) |
|  | 1. When child is doing something, does he/she finish it? | 3692 (49.3) | 0 (0.0) |
|  | 1. Does child have to be told several times so that then he/she can finish what he/she is doing?* | 4258 (56.8) | 0 (0.0) |
|  | 1. When child is doing something, does he/she lose focus easily?* | 3912 (52.5) | 0 (0.0) |
| Approaches to Learning | 1. Does child show any sign of interest to learn new things? | 6550 (87.4) | 0 (0.0) |
|  | 1. Does child try to learn how to play with new toys? | 6230 (83.1) | 0 (0.0) |
|  | 1. Has child ever used any object to role play (using imagination) e.g. banana stem horse, sword fighting etc? | 4832 (64.5) | 0 (0.0) |
|  | 1. Does child show interest in playing games such as collecting pebbles, jumping with rubber bands, hide and seek, ball throwing etc? | 5562 (74.2) | 0 (0.0) |
|  | 1. When the kids go somewhere with you or another household member, are they brave to survey or ask some questions with another person? | 4874 (65.0) | 0 (0.0) |
|  | 1. When child is doing a particular activity, they will pay intense attention to the activity. | 4862 (62.5) | 0 (0.0) |
|  | 1. Does child think things out before starting a task? | 2613 (34.9) | 0 (0.0) |
| Numeracy | 1. Can child distinguish between a triangle, circle and rectangle? | 1203 (16.1) | 0 (0.0) |
|  | 1. Can child tell at least three different colours? | 4345 (58.0) | 0 (0.0) |
|  | 1. Can child distinguish objects based on shape, colour, and size? | 2050 (27.4) | 0 (0.0) |
|  | 1. Can child count from 1 to 10? | 2900 (38.7) | 0 (0.0) |
|  | 1. Can child count from 1 to 20? | 1320 (17.6) | 0 (0.0) |
|  | 1. Does child know that a tiger is taller than a cat?' | 2527 (33.7) | 0 (0.0) |
|  | 1. Does child know morning, afternoon and evening? | 5623 (75.0) | 0 (0.0) |
|  | 1. Can child tell you if it is yesterday, today or tomorrow? | 4813 (64.2) | 0 (0.0) |
|  | 1. Does child know that an elephant is heavier than a pig? | 1919 (25.6) | 0 (0.0) |
|  | 1. Does child know that 8 is more than 2? | 1043 (13.9) | 0 (0.0) |
| Reading | 1. Can child read in the correct direction from left to right and from top to bottom (even if they can’t read)? | 1288 (17.2) | 0 (0.0) |
|  | 1. Can child tell at least 3 letters of the alphabet? | 2070 (27.6) | 0 (0.0) |
|  | 1. Can child tell at least 10 letters of the alphabet? | 963 (12.9) | 0 (0.0) |
|  | 1. Can child read at least 4 simple words? | 376 (5.0) | 0 (0.0) |
|  | 1. Can child read difficult words such as axe, buffalo? | 323 (4.3) | 0 (0.0) |
|  | 1. Can child read simple sentences? | 314 (4.2) | 0 (0.0) |
| Writing | 1. Can child write or draw with pencil, coloured pencils or pen? | 4632 (61.8) | 0 (0.0) |
|  | 1. Can child draw a picture that you can tell what it is? | 993 (13.3) | 0 (0.0) |
|  | 1. Can child write at least three letters such as A, B, C? | 1236 (16.5) | 0 (0.0) |
|  | 1. Can child write his/her own name? | 310 (4.1) | 0 (0.0) |
|  | 1. Can child write a simple word? | 239 (3.2) | 0 (0.0) |
|  | 1. Can child write a simple sentence? | 135 (1.8) | 0 (0.0) |

*Note.* * = reverse scored items.
