# Supplementary material for: Measuring early childhood development in multiple contexts: the internal factor structure and reliability of the early Human Capability Index in seven low and middle income countries
Source: BMC Pediatr. 2019 Dec 3;19:471. doi: 10.1186/s12887-019-1852-5 (PMC6889461; doi:10.1186/s12887-019-1852-5)
Supplement: Supplementary file 5 — Additional file 5: Table S5. Samoa eHCI items and n (%) children for whom their caregiver/ teacher reported yes/able. [file 12887_2019_1852_MOESM5_ESM.docx]

**Supplementary Table 5.** Samoa eHCI items and n (%) children for whom their caregiver/teacher reported yes/able

| Domain | Item | Yes/Able | | Missing |
| --- | --- | --- | --- | --- |
| Physical  Health | 1. Is the child frequently sick?* | 1076 (8.8) | | 0 (0.0) |
|  | 1. Does the child have good hygiene (e.g. always washes hands after toileting)? | 9503 (78.0) | | 0 (0.0) |
|  | 1. Does the child know the difference between healthy and unhealthy food? | 4757 (39.0) | | 0 (0.0) |
| Verbal Communication | 1. Does the child use a group of words in conversation? | 6955 (57.1) | | 0 (0.0) |
|  | 1. Can the child talk about something that they have done? | 7895 (64.8) | | 0 (0.0) |
|  | 1. Can the child use respectful language? | 4618 (37.9) | | 0 (0.0) |
|  | 1. Does the child always ask questions? | 7980 (65.5) | | 0 (0.0) |
| Cultural Knowledge | 1. Does the child show respect and compassion to others? | 4597 (37.7) | | 0 (0.0) |
|  | 1. Can the child name two Samoan foods? | 4960 (40.7) | | 0 (0.0) |
|  | 1. Can the child give the names of two plants? | 4809 (39.4) | | 0 (0.0) |
|  | 1. Can the child show the Samoan cultural values of humility? | 5311 (43.6) | | 0 (0.0) |
|  | 1. Do you believe and trust that this child can do anything that you ask them to do? | 4304 (35.3) | | 0 (0.0) |
|  | 1. Does the child do sharing in relationships and work co-operatively with others? | 4920 (40.4) | | 0 (0.0) |
|  | 1. Does the child participate in community cultural routines (e.g. sports and entertainment)? | 5118 (42.0) | | 0 (0.0) |
|  | 1. Can the child say a short prayer? | 7090 (58.2) | | 0 (0.0) |
| Social and Emotional | 1. Is the child happy to share toys and belongings? | 7189 (59.0) | | 0 (0.0) |
|  | 1. Does the child take care of their own belongings? | 6348 (52.1) | | 0 (0.0) |
|  | 1. Does the child show respect to adults? | 4762 (39.1) | | 0 (0.0) |
|  | 1. Does the child show respect to children? | 4724 (38.7) | | 0 (0.0) |
|  | 1. Does the child accept decision making for their actions? | 5344 (43.8) | | 0 (0.0) |
|  | 1. If you discipline the child on appropriate behaviour, do they learn or not? | 5257 (43.1) | | 0 (0.0) |
|  | 1. Is the child considerate of other people’s feelings? | 4620 (37.9) | | 0 (0.0) |
|  | 1. Is the child always helpful? | 7449 (61.1) | | 0 (0.0) |
|  | 1. Does the child have friends? | 10113 (83.0) | | 0 (0.0) |
|  | 1. Is the child playful and undisciplined?* | 9602 (78.8) | | 0 (0.0) |
|  | 1. Is the child impatient?* | 6111 (50.1) | | 0 (0.0) |
|  | 1. Does the child understand the difference between right and wrong? | 5514 (45.2) | | 0 (0.0) |
|  | 1. Does the child follow simple commands? | 8396 (68.9) | | 0 (0.0) |
| Perseverance | 1. Can the child perform tasks independently? | | 4295 (35.2) | 0 (0.0) |
|  | 1. Does the child always do tasks completely? | | 4216 (34.6) | 0 (0.0) |
|  | 1. Is the child usually ordered to complete tasks?* | | 7221 (59.2) | 0 (0.0) |
|  | 1. Does the child lose interest easily and give up?* | | 8228 (67.5) | 0 (0.0) |
| Approaches to Learning | 1. Does the child show more curiosity about something new in comparison to something familiar? | | 8382 (68.8) | 0 (0.0) |
|  | 1. Is the child happy to explore and investigate the function of his/her toys? | | 8443 (69.3) | 0 (0.0) |
|  | 1. Does the child always want to learn new things? | | 8171 (67.0) | 0 (0.0) |
|  | 1. When in an unfamiliar environment with a familiar person present, does the child feel free to explore? | | 6839 (56.1) | 0 (0.0) |
|  | 1. Is the child diligent in the tasks that you ask him/her to do? | | 5022 (41.2) | 0 (0.0) |
| Numeracy | 1. Can the child recognise geometric shapes (e.g. triangle, circle, square)? | | 4259 (34.9) | 0 (0.0) |
|  | 1. Can the child observe and identify three colours? | | 4580 (37.6) | 0 (0.0) |
|  | 1. Can the child sort and classify objects in common characteristics of colours, shapes and sizes? | | 3466 (28.4) | 0 (0.0) |
|  | 1. Can the child name and recognise the symbols of all numbers from 1-10? | | 5088 (41.7) | 0 (0.0) |
|  | 1. Can the child count to 10? | | 8255 (67.7) | 0 (0.0) |
|  | 1. Can the child count to 20? | | 2630 (21.6) | 0 (0.0) |
|  | 1. Can the child count to 100? | | 679 (5.6) | 0 (0.0) |
|  | 1. Does the child know that a horse is taller than a dog? | | 5141 (42.2) | 0 (0.0) |
|  | 1. Does the child know the order of the day (morning, afternoon, then evening)? | | 3022 (24.8) | 0 (0.0) |
|  | 1. Does the child understand the concepts of yesterday, today and tomorrow? | | 3102 (25.4) | 0 (0.0) |
|  | 1. Does the child know that a bus weighs more than a bicycle? | | 4322 (35.5) | 0 (0.0) |
|  | 1. Does the child know that number 8 is bigger than number 2? | | 3900 (32.0) | 0 (0.0) |
| Reading | 1. Does the child know the sounds of 3 letters of the Samoan alphabet? | | 6563 (53.8) | 0 (0.0) |
|  | 1. Can the child identify at least 3 letters of the Samoan alphabet? | | 6119 (50.2) | 0 (0.0) |
|  | 1. Can the child identify at least 10 letters of the Samoan alphabet? | | 4294 (35.2) | 0 (0.0) |
|  | 1. Can the child hold a book and turn the pages in the right way? | | 4592 (37.7) | 0 (0.0) |
|  | 1. Can the child follow the right reading direction (e.g. left to right, top to bottom)? | | 3148 (25.8) | 0 (0.0) |
|  | 1. Can the child read simple and popular words? | | 3296 (27.0) | 0 (0.0) |
| Writing | 1. Can the child draw something that is identifiable (e.g. a stick person)? | | 5467 (44.8) | 0 (0.0) |
|  | 1. Can the child copy or trace the shape of a letter? (e.g. A, E, I) | | 5178 (42.5) | 0 (0.0) |
|  | 1. Can the child write 3 letters? (e.g. A, E, I) | | 5028 (41.2) | 0 (0.0) |
|  | 1. Can the child write their own name? | | 3228 (26.5) | 0 (0.0) |
|  | 1. Can the child write simple words? | | 3334 (27.3) | 0 (0.0) |

*Note.* * = reverse scored items.
