# Supplementary material for: Measuring early childhood development in multiple contexts: the internal factor structure and reliability of the early Human Capability Index in seven low and middle income countries
Source: BMC Pediatr. 2019 Dec 3;19:471. doi: 10.1186/s12887-019-1852-5 (PMC6889461; doi:10.1186/s12887-019-1852-5)
Supplement: Supplementary file 6 — Additional file 6: Table S6. Tuvalu eHCI items and n (%) children for whom their caregiver/ teacher reported yes/able. [file 12887_2019_1852_MOESM6_ESM.docx]

**Supplementary Table 6.** Tuvalu eHCI items and n (%) children for whom their caregiver/teacher reported yes/able

| Domain | Item | Yes/Able | Missing |
| --- | --- | --- | --- |
| Physical  Health | 1. Does this child get sick often?* | 219 (39.9) | 3 (0.5) |
|  | 1. Is the child practicing cleanliness and healthy living (clean hands each time he/she uses the toilet)? | 440 (80.1) | 0 (0.0) |
|  | 1. Is the child personally practicing cleanliness and healthy living on his own? | 337 (61.4) | 0 (0.0) |
|  | 1. Is the child being careful from being hurt (burnt, drown, fall, stumble)? | 427 (77.8) | 3 (0.5) |
|  | 1. Does the child know the difference between good and bad food? | 433 (78.9) | 0 (0.0) |
| Verbal Communication | 1. The child is able to use a group of words. | 488 (88.9) | 0 (0.0) |
|  | 1. The child is able to use a complete sentence. | 346 (63.0) | 0 (0.0) |
|  | 1. The child is able to wait for the other person to finish speaking, in a conversation, before he/she could speak. | 263 (47.9) | 0 (0.0) |
|  | 1. The child is able to interpret things in Tuvaluan. | 351 (63.9) | 0 (0.0) |
|  | 1. The child is able to communicate as a mature person (talkative, enquiring). | 378 (68.9) | 0 (0.0) |
|  | 1. The child knows his/her name. | 534 (97.3) | 1 (0.2) |
|  | 1. The child knows the name of one of his/her parents/guardians. | 527 (96.0) | 0 (0.0) |
| Cultural Knowledge | 1. The child is able to exhibit behaviours of affection, understanding and patience to others. | 364 (66.3) | 0 (0.0) |
|  | 1. The child is able to identify two valuable foods in Tuvalu. | 381 (69.4) | 0 (0.0) |
|  | 1. The child is able to identify two edible plants in Tuvalu. | 398 (72.5) | 1 (0.2) |
|  | 1. The child is able to express Tuvaluan behaviours and traditions (giving respect to others, being humble). | 277 (50.5) | 1 (0.2) |
|  | 1. The child is able to exhibit behaviours of loyalty and commitment to do something. | 312 (56.8) | 0 (0.0) |
|  | 1. The child is able to demonstrate qualities of good friendship. | 448 (81.6) | 0 (0.0) |
|  | 1. The child is able to join cultural and traditional way of Tuvalu life (Tuvaluan local dance). | 408 (74.3) | 0 (0.0) |
|  | 1. The child is able to say a usual short prayer. | 474 (86.3) | 0 (0.0) |
|  | 1. The child is able to say a short prayer using own words. | 232 (42.3) | 0 (0.0) |
| Social and Emotional | 1. The child is willing to share his toys and belongings with others. | 432 (78.7) | 0 (0.0) |
|  | 1. The child is able to keep his belongings very well. | 377 (68.7) | 0 (0.0) |
|  | 1. The child knows how to respect older people. | 357 (65.0) | 0 (0.0) |
|  | 1. The child knows how to respect other children. | 334 (60.8) | 0 (0.0) |
|  | 1. The child accepts his/her responsibilities when he/she is being instructed to carry them out. | 435 (79.2) | 0 (0.0) |
|  | 1. The child welcomes the opinions of others. | 341 (62.1) | 0 (0.0) |
|  | 1. The child continually does whatever was told of him/her not to do.* | 343 (62.5) | 0 (0.0) |
|  | 1. The child is willing to help others. | 485 (88.3) | 1 (0.2) |
|  | 1. The child gets along easily with other children. | 460 (83.8) | 0 (0.0) |
|  | 1. The child frequently kicks, bites, or hits older children or people.* | 207 (37.7) | 1 (0.2) |
|  | 1. The child can be patient long enough before receiving his/her needs. | 333 (60.7) | 1 (0.2) |
|  | 1. The child always knows the difference between good and bad. | 357 (65.0) | 0 (0.0) |
|  | 1. The child can follow simple instructions. | 519 (94.5) | 0 (0.0) |
| Perseverance | 1. The child can always do his/her work on his own. | 411 (74.9) | 1 (0.2) |
|  | 1. The child always completes his/her work. | 318 (57.9) | 1 (0.2) |
|  | 1. The child always needs to be reminded about completing what he/she was doing.* | 402 (73.2) | 1 (0.2) |
|  | 1. The child gets bored quickly when he/she was doing his/her job task.* | 368 (67.0) | 1 (0.2) |
| Approaches to Learning | 1. The child prefers learning new ideas to familiar concepts. | 499 (90.9) | 1 (0.2) |
|  | 1. The child examines how a new toy works. | 495 (90.2) | 1 (0.2) |
|  | 1. The child always desires learning of new concepts. | 495 (90.2) | 1 (0.2) |
|  | 1. When the child is placed in an unfamiliar setting with a person he/she knows, would he be delighted to learn? | 424 (77.2) | 1 (0.2) |
|  | 1. The child always considers a school activity carefully and works on it wholeheartedly. | 368 (67.0) | 1 (0.2) |
| Numeracy | 1. The child is able to see shapes such as a triangle, a circle, and a square. | 338 (61.6) | 1 (0.2) |
|  | 1. The child is able to pronounce names, and divide 3 colours of more. | 336 (61.2) | 1 (0.2) |
|  | 1. The child is able to divide and arrange these items to their own parts (such as shapes, colours and sizes). | 332 (60.5) | 1 (0.2) |
|  | 1. The child is able to pronounce and recognise numbers from 1 to 10. | 316 (57.6) | 1 (0.2) |
|  | 1. The child is able to count up to 10. | 498 (90.7) | 1 (0.2) |
|  | 1. The child is able to count up to 20. | 224 (40.8) | 1 (0.2) |
|  | 1. The child is able to count up to 100. | 62 (11.3) | 2 (0.4) |
|  | 1. The child is aware that the dog is taller than the mouse. | 410 (74.7) | 1 (0.2) |
|  | 1. The child is aware of the order of time in a day (morning, then afternoon then evening). | 253 (46.1) | 1 (0.2) |
|  | 1. The child is aware of yesterday, today and tomorrow. | 189 (34.4) | 1 (0.2) |
|  | 1. The child is aware that the chair is heavier than a pencil. | 383 (69.8) | 0 (0.0) |
|  | 1. The child is aware that the number 8 is larger than the number 2. | 239 (43.5) | 2 (0.4) |
| Reading | 1. The child knows the pronunciation of three letters in the sequence A E I. | 399 (72.7) | 1 (0.2) |
|  | 1. The child is able to identify 3 letters or more in the sequence A E I. | 347 (63.2) | 1 (0.2) |
|  | 1. The child is able to identify 10 letters or more in the sequence A E I. | 210 (38.3) | 1 (0.2) |
|  | 1. The child is able to properly hold the book and appropriately turn its pages in the right order. | 308 (56.1) | 1 (0.2) |
|  | 1. The child is able to follow the right way or reading (from left to right, from top to bottom). | 232 (42.3) | 1 (0.2) |
|  | 1. The child is able to read 4 or more familiar words. | 160 (29.1) | 1 (0.2) |
| Writing | 1. The child is able to draw a picture that could be recognised (person's image). | 439 (80.0) | 1 (0.2) |
|  | 1. Can this child copy or trace the outline of a letter over an already written letter? | 364 (66.3) | 1 (0.2) |
|  | 1. The child is able to write 3 letters or more (A, E, I). | 282 (51.4) | 1 (0.2) |
|  | 1. The child is able to write his name. | 237 (43.2) | 1 (0.2) |
|  | 1. The child is able to write simple words. | 184 (33.5) | 4 (0.7) |

*Note.* * = reverse scored items.
