# Supplementary material for: Measuring early childhood development in multiple contexts: the internal factor structure and reliability of the early Human Capability Index in seven low and middle income countries
Source: BMC Pediatr. 2019 Dec 3;19:471. doi: 10.1186/s12887-019-1852-5 (PMC6889461; doi:10.1186/s12887-019-1852-5)
Supplement: Supplementary file 8 — Additional file 8: Table S8. Factor loadings from confirmatory factor analysis in China. [file 12887_2019_1852_MOESM8_ESM.docx]

**Supplementary Table 8.** Factor loadings from confirmatory factor analysis in China

|  | F10 | F2 | F3 | F4 | F5 | F6 | F7 | F8 | F9 |
| --- | --- | --- | --- | --- | --- | --- | --- | --- | --- |
| Phys 1 | 0.12 |  |  |  |  |  |  |  |  |
| Phys 2 | 0.71 |  |  |  |  |  |  |  |  |
| Phys 3 | 0.64 |  |  |  |  |  |  |  |  |
| Comm 1 |  | 0.52 |  |  |  |  |  |  |  |
| Comm 2 |  | 0.86 |  |  |  |  |  |  |  |
| Comm 3 |  | 0.90 |  |  |  |  |  |  |  |
| Comm 4 |  | 0.74 |  |  |  |  |  |  |  |
| Comm 5 |  | 0.70 |  |  |  |  |  |  |  |
| Comm 6 |  | 0.78 |  |  |  |  |  |  |  |
| Cult 1 |  |  | 0.71 |  |  |  |  |  |  |
| Cult 2 |  |  | 0.79 |  |  |  |  |  |  |
| Cult 3 |  |  | 0.62 |  |  |  |  |  |  |
| Cult 4 |  |  | 0.79 |  |  |  |  |  |  |
| Cult 5 |  |  | 0.84 |  |  |  |  |  |  |
| Cult 6 |  |  | 0.84 |  |  |  |  |  |  |
| Cult 7 |  |  | 0.80 |  |  |  |  |  |  |
| Soc 1 |  |  |  | 0.67 |  |  |  |  |  |
| Soc 2 |  |  |  | 0.67 |  |  |  |  |  |
| Soc 3 |  |  |  | 0.79 |  |  |  |  |  |
| Soc 4 |  |  |  | 0.80 |  |  |  |  |  |
| Soc 5 |  |  |  | 0.74 |  |  |  |  |  |
| Soc 6 |  |  |  | 0.77 |  |  |  |  |  |
| Soc 7 |  |  |  | 0.59 |  |  |  |  |  |
| Soc 8 |  |  |  | 0.14 |  |  |  |  |  |
| Soc 9 |  |  |  | 0.04 |  |  |  |  |  |
| Soc 10 |  |  |  | 0.68 |  |  |  |  |  |
| Persev 1 |  |  |  |  | 0.82 |  |  |  |  |
| Persev 2 |  |  |  |  | 0.83 |  |  |  |  |
| Persev 3 |  |  |  |  | 0.05 |  |  |  |  |
| Persev 4 |  |  |  |  | 0.02 |  |  |  |  |
| Appr 1 |  |  |  |  |  | 0.65 |  |  |  |
| Appr 2 |  |  |  |  |  | 0.70 |  |  |  |
| Appr 3 |  |  |  |  |  | 0.73 |  |  |  |
| Appr 4 |  |  |  |  |  | 0.59 |  |  |  |
| Appr 5 |  |  |  |  |  | 0.80 |  |  |  |
| Appr 6 |  |  |  |  |  | 0.81 |  |  |  |
| Num 1 |  |  |  |  |  |  | 0.76 |  |  |
| Num 2 |  |  |  |  |  |  | 0.79 |  |  |
| Num 3 |  |  |  |  |  |  | 0.78 |  |  |
| Num 4 |  |  |  |  |  |  | 0.89 |  |  |
| Num 5 |  |  |  |  |  |  | 0.89 |  |  |
| Num 6 |  |  |  |  |  |  | 0.90 |  |  |
| Num 7 |  |  |  |  |  |  | 0.77 |  |  |
| Num 8 |  |  |  |  |  |  | 0.69 |  |  |
| Num 9 |  |  |  |  |  |  | 0.74 |  |  |
| Num 10 |  |  |  |  |  |  | 0.76 |  |  |
| Num 11 |  |  |  |  |  |  | 0.73 |  |  |
| Num 12 |  |  |  |  |  |  | 0.86 |  |  |
| Read 1 |  |  |  |  |  |  |  | 0.94 |  |
| Read 2 |  |  |  |  |  |  |  | 0.88 |  |
| Read 3 |  |  |  |  |  |  |  | 0.47 |  |
| Read 4 |  |  |  |  |  |  |  | 0.77 |  |
| Read 5 |  |  |  |  |  |  |  | 0.84 |  |
| Read 6 |  |  |  |  |  |  |  | 0.75 |  |
| Read 7 |  |  |  |  |  |  |  | 0.81 |  |
| Read 8 |  |  |  |  |  |  |  | 0.73 |  |
| Writ 1 |  |  |  |  |  |  |  |  | 0.66 |
| Writ 2 |  |  |  |  |  |  |  |  | 0.89 |
| Writ 3 |  |  |  |  |  |  |  |  | 0.69 |

*Note*. Phys=Physical Health, Comm=Verbal Communication, Cult=Cultural Knowledge, Soc=Social and Emotional, Persev=Perseverance, Appr=Approaches to Learning, Num=Numeracy, Read=Reading, and Writ=Writing.
